# A novel method of selective removal of human DNA improves PCR sensitivity for detection of *Salmonella* Typhi in blood samples

**DOI:** 10.1186/1471-2334-12-164

**Published:** 2012-07-27

**Authors:** Liqing Zhou, Andrew J Pollard

**Affiliations:** 1 Oxford Vaccine Centre, Department of Paediatrics, University of Oxford, Oxford, UK; 2 Current address: Novartis Vaccines and Diagnostics s.r.l., via Fiorentina 1, 53100, Siena, Italy

**Keywords:** Typhoid, *Salmonella* Typhi, PCR detection

## Abstract

**Background:**

Enteric fever is a major public health problem, causing an estimated 21million new cases and 216,000 or more deaths every year. Current diagnosis of the disease is inadequate. Blood culture only identifies 45 to 70% of the cases and is time-consuming. Serological tests have very low sensitivity and specificity. Clinical samples obtained for diagnosis of enteric fever in the field generally have <1 organism/ml of blood, so that even PCR-based methods, widely used for detection of other infectious diseases, are not a straightforward option in typhoid diagnosis. We developed a novel method to enrich target bacterial DNA by selective removal of human DNA from blood samples, enhancing the sensitivity of PCR tests. This method offers the possibility of improving PCR assays directly using clinical specimens for diagnosis of this globally important infectious disease.

**Methods:**

Blood samples were mixed with ox bile for selective lysis of human blood cells and the released human DNA was then digested with addition of bile resistant micrococcal nuclease. The intact *Salmonella* Typhi bacteria were collected from the specimen by centrifugation and the DNA extracted with QIAamp DNA mini kit. The presence of *Salmonella* Typhi bacteria in blood samples was detected by PCR with the *fliC-d* gene of *Salmonella* Typhi as the target.

**Results:**

Micrococcal nuclease retained activity against human blood DNA in the presence of up to 9% ox bile. Background human DNA was dramatically removed from blood samples through the use of ox bile lysis and micrococcal nuclease for removal of mammalian DNA. Consequently target *Salmonella* Typhi DNA was enriched in DNA preparations and the PCR sensitivity for detection of *Salmonella* Typhi in spiked blood samples was enhanced by 1,000 fold.

**Conclusions:**

Use of a combination of selective ox-bile blood cell lysis and removal of human DNA with micrococcal nuclease significantly improves PCR sensitivity and offers a better option for improved typhoid PCR assays directly using clinical specimens in diagnosis of this globally important infection disease which we believe could be of importance in improving clinical care and providing effective evaluation of novel vaccines.

## Background

There are an estimated 21million new cases and 216,000 deaths attributed to typhoid fever every year [1]. The disease, caused by *Salmonella enterica* serovar Typhi, remains a common problem in many parts of the world where access to clean water is limited. In the regions where enteric fever is common, clinical diagnosis of typhoid fever is inadequate, as the symptoms it causes are non-specific and overlap with those of many other febrile illness including malaria, dengue fever, rickettsioses, leptospirosis and melioidosis [2]. Baker *et al.* has recently presented the current position in typhoid diagnostics, highlighting the need for technological improvements and potential future approaches [3].

The first typhoid diagnostic, the Widal test, was developed in 1896 and is still widely used. The Widal test is dependent on agglutination in an assay in which *Salmonella* Typhi cells are used to detect antibodies in blood. Many of the surface antigens of the *Enterobacteriaceae* of which *Salmonella* Typhi is a member, demonstrate significant conservation and induce antibodies that are cross-reactive. Consequently, the Widal test has very low sensitivity and specificity, and little or no practical value in endemic areas despite its continued use [4]. Several other serologically based assays are available for use in typhoid diagnosis including Typhidot and Tubex [5,6], but have the same problems associated with the use of the Widal test. When assessed in population-based typhoid surveillance studies in several countries and in all locations Tubex and Typhidot had the sensitivity and specificity of only around 70% and 80% respectively [7,8].

Isolation of the causative organism remains the most reliable diagnostic method in suspected typhoid fever and blood has been the main sample used for culture of the organism since 1900 [9,10]. However, blood culture can only identify 45 to 70% of patients with typhoid fever, and is highly dependent on the amount of blood sampled. In addition the bacteraemic level of *Salmonella* Typhi, the presence of bactericidal activity in the blood, recent administration of antibiotics, the type of culture medium used, and the length of incubation period may all affect the sensitivity [11,12]. The intracellular nature of *Salmonella* serovar Typhi also slows its growth in blood culture media. One study found that more than 50% of bacterial cells were present intracellularly in the blood from patients with typhoid fever [11]. In addition, blood culture facilities are rare in many developing countries, often limited only to major hospitals in large cities, making access to blood culture facilities a major limiting factor in typhoid diagnosis. Furthermore, blood culture takes at least 2 to 5 days before the identification of the organism, which is often too late to initiate appropriate antibiotic therapy.

Given the problems associated with serological methods and blood culture, PCR based methods have been exploited recently because they can theoretically amplify DNA only from *Salmonella* Typhi (specificity) and should detect even low numbers of live or dead bacterial cells (sensitivity). Many *Salmonella* Typhi PCR-based assays have targeted the *fliC-d* gene, utilizing nested primers to improve sensitivity [13-18] and reported excellent sensitivity and specificity when compared to positive cases (blood culture proven) and healthy controls. However, the number of *Salmonella* bacteria circulating in the blood of a patient with bacteremia is generally low with the majority of patients having <1 organism/ml of blood [19]. This means that the PCR template in clinical preparations is dominated by human DNA and could cause false-positive PCR signals due to the non-specific binding of primers and false-negative results due to reduced sensitivity. In practice, the large excess of human DNA does indeed cause problems for PCR-based pathogen detection in blood, particularly in samples with low bacterial numbers [20,21]. Furthermore, small volumes of blood are often used for DNA extraction or as template in the PCR, which will significantly lower the sensitivity of these tests. A DNA or bacterial capture system or even a culture enrichment step prior to amplification may improve molecular sensitivity of PCR based assays. We have recently reported a fast and highly sensitive blood culture-PCR method for detection of *Salmonella* Typhi [22].

**Figure 1:**
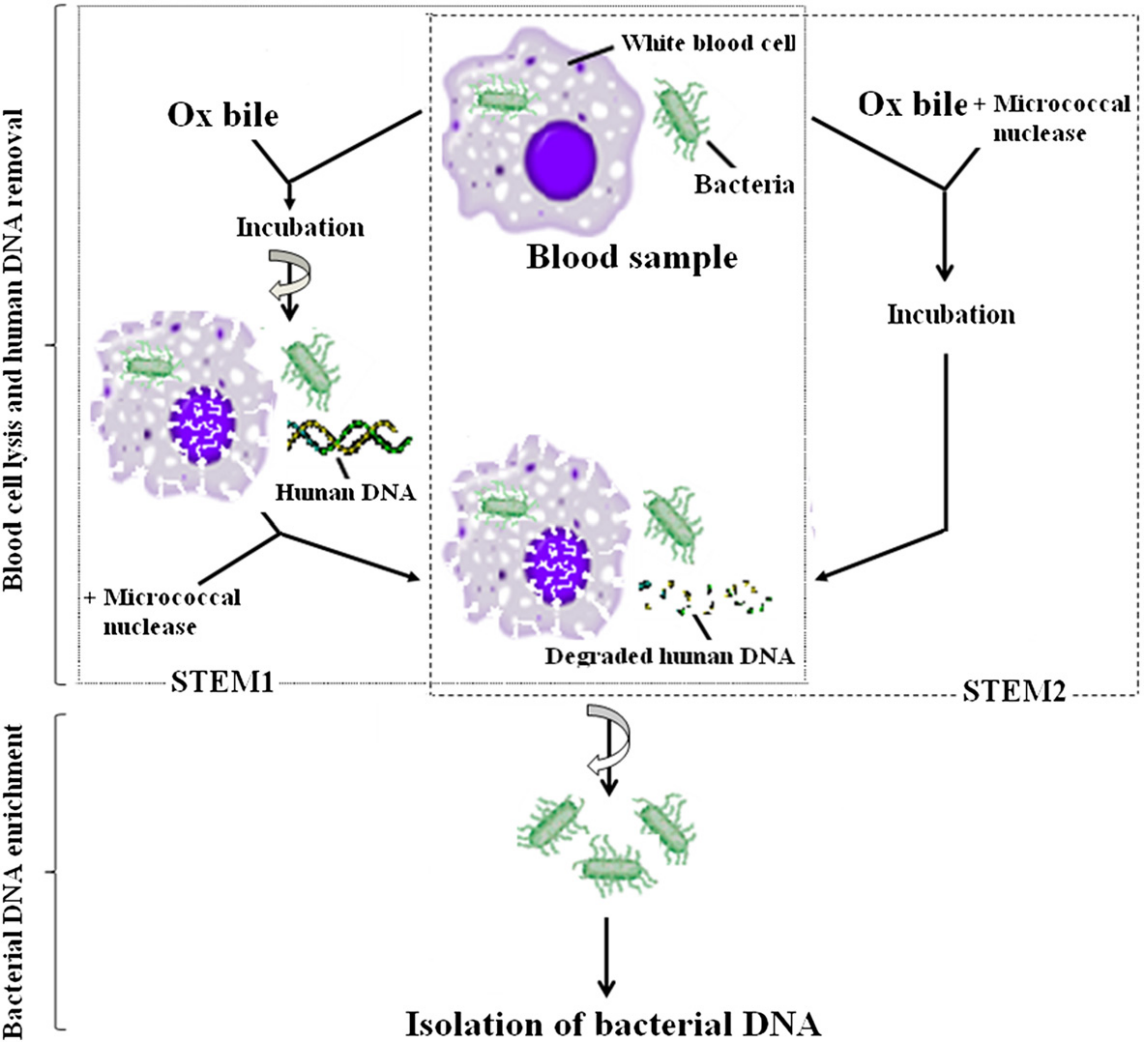
The format and principle of the selective target DNA enrichment method.

From the discussion above it is clear that an optimised and sensitive assay for PCR diagnosis of typhoid would work directly on clinical samples, release intracellular bacteria to improve access to the bacteria DNA, and finally reduce background human DNA to enrich the target bacterial DNA. This can be achieved by using a combination of selective detergents and nucleases. Ox bile can selectively lyze blood cells, releasing the human DNA which is accessible to enzymatic degradation. Due to its bile-resistance, *Salmonella* Typhi cells remain intact and are collected by centrifugation for the extraction of pure *Salmonella* Typhi DNA. Micrococcal nuclease, derived from S*taphylococcus aureus,* is a relatively non-specific endo-exonuclease and can degrade both DNA and RNA to 3´ phosphomononucleotides and dinucleotides in the pH range of 7.0-10.0. The enzyme activity is strictly dependent on Ca^2+^ and therefore is easily inactivated by EGTA. We investigated further micrococcal nuclease activity in ox bile and describe herein a novel method for the selective enrichment of *Salmonella* Typhi DNA from whole blood samples using ox bile and micrococcal nuclease, which we have termed “the selective target DNA enrichment method” (STEM). This method does not require blood culture. By applying STEM, we could show a great reduction of the human background DNA in the DNA preparations from blood samples, and consequently the sensitivity in PCR detection of *Salmonella* Typhi in blood samples is dramatically enhanced. Figure 1 shows the format and principle of STEM.

## Materials and methods

### Strains and culture

Wild-type *Salmonella* serovar Typhi Quailes strain, obtained from the University of Maryland, was used to spike blood samples in this study. The strain was sub-cultured in tryptone soya broth (TSB) or on tryptone soya agar (TSA) (Oxoid, Basingstoke, UK) as needed.

### Preparation of spiked blood samples

A colony of overnight culture of *Salmonella* Typhi Quailes strain on TSA was inoculated into 2 ml TSB, and cultured in a 37°C incubator with shaking at 200 revolutions per minute (RPM) for about 2 hours. The culture was first adjusted to an optical density at 600nm of 0.25 and then prepared for serial 10-fold dilutions in TSB. An aliquot of 100 μl each of dilutions was plated onto a TSA plate, and the plates were incubated overnight at 37°C to determine the colony forming unit (CFU) of *Salmonella* Typhi per millilitre. Blood was obtained from healthy individuals with a sterile syringe and immediately pipetted into tubes containing heparin. 100 μl each of the bacterial dilutions was spiked into a 900 μl blood sample and 200 μl spiked blood samples were used for DNA isolation.

The blood samples used in the study were obtained from laboratory volunteers with written informed consent, in accordance with the university policies - Taking Blood Samples from Colleagues or Students for Research and Teaching OHS Policy Document 1/03 approved by the Central University Research Ethics Committee (CUREC), University of Oxford.

### Conventional DNA preparation

Blood DNA was isolated from 200 μl heparinised blood sample using QIAamp DNA mini kit (Qiagen, Crawley, UK) according to the manufacturer’s instruction, except that the DNA was eluted with 50 μl Buffer AE and incubated at 65°C for 5 minutes before centrifugation. The DNA was stored at −20°C until further use. The aliquots of DNA preparation were run on a 1% agarose gel, stained with ethidium bromide and visualized by UV transllumination. The DNA concentration was determined by UV spectrometry.

### Digestion of human blood DNA with micrococcal nuclease

0.5 μg human blood DNA was mixed with 10^3^ gel units of micrococcal nuclease (New England Biolab, Herts, UK) in 20 μl reaction volume containing 1 μM CaCl_2_ and 0, 1%, 3%, 5%, 7% or 9% ox bile. Following incubation at room temperature for 10 minutes, the reaction mixture was run on a 1% agarose gel, stained with ethidium bromide, and photographed under UV illuminator.

### Selective *Salmonella* Typhi target DNA enrichment and preparation

A flow diagram of two selective target DNA enrichment methods (STEM1 and STEM2) used in this study for enrichment of *Salmonella* Typhi bacterial DNA from spiked blood samples was shown in Figure 1.

In STEM1, 200 μl spiked blood sample was gently mixed with equal volume of 10% ox bile (Oxgall, BD Biosciences, Oxford, UK), incubated at room temperature for 10 minutes, and then centrifuged at 13,000 RPM for 5 minutes. The pellet was re-suspended in 100 μl 0.1 μM CaCl_2_, and 1 μl (10^3^ gel units) micrococcal nuclease was added. The mixture was incubated at 37°C for 10 minutes and then centrifuged again at 13,000 RPM for 5 minutes. The pellet contained the enriched *Salmonella* Typhi bacteria and was re-suspended in 200 μl phosphate buffered saline (PBS) for bacterial DNA isolation.

In STEM2, 200 μl spiked blood was mixed together with 200 μl of 10% ox bile, 4 μl of 0.1 mM CaCl_2_ and 2 μl (2×10^3^ gel units) micrococcal nuclease, incubated at room temperature for 20 minutes, and then centrifuged at 13,000 RPM for 5 minutes to collect the pellet containing enriched bacterial cells. The pellet was re-suspended in 200 μl PBS for bacterial DNA isolation.

Following the re-suspension of the enriched bacterial pellet obtained in STEM1 or STEM2, bacterial DNA was isolated using QIAamp DNA mini kit in the same way as described in the section of Conventional DNA preparation above, except that the mixture of 20 μl proteinase K solution and bacterial pellet suspension was incubated for 5 minutes at room temperature to completely remove any residual activity of micrococcal nuclease before Buffer AL was added.

### PCR primers of *Salmonella s*erovar Typhi Quailes strain

The PCR primers for *Salmonella* serovar Typhi Quailes strain were designed according to the *fliC-d* gene sequence of *Salmonella* serovar Typhi (Accession number L21912): H-for (ACTCAGGCTTCCCGTAACGC) and Hd-rev (GGCTAGTATTGTCCTTATCGG) [15], and synthesized by Sigma Genosys (Sigma-Aldrich, Dorset, England).

### PCR protocol

The PCR reaction was carried out in a 50 μl volume, comprising 0.5 U of Taq DNA polymerase (Qiagen, Crawley, UK), 1 x Qiagen PCR buffer, 1.5 mM magnesium chloride, 200 μM concentrations of each deoxynucleoside triphosphate, 0.5 μM concentrations of the primers H-for and Hd-rev, and 10 μl of DNA template. The following amplification steps were used: 1 cycle of 95°C for 5 min; 35 cycles of 93°C for 30 sec, 55°C for 30 sec, and 72°C for 40 sec; and 1 cycle of 72°C for 5 min. The PCR amplification product was separated by electrophoresis on a 1% agarose gel, stained with ethidium bromide, and photographed by a UV transilluminator.

## Results

### Micrococcal nuclease is active on human DNA in the presence of ox bile

**Figure 2:**
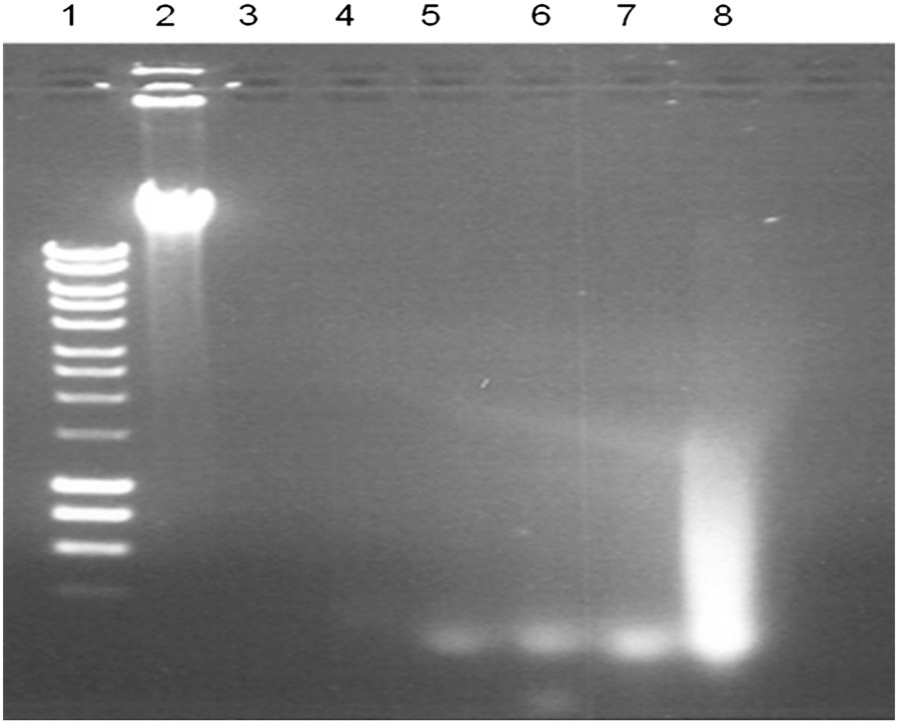
**Digestion of human blood DNA with micrococcal nuclease at different concentration of ox bile.** Lane 1: DNA molecular marker; Lane 2: No micrococcal nuclease and no ox bile; Lanes 3–8: 0%, 1%, 3%, 5%, 7% and 9% ox bile concentration respectively.

Human blood DNA was incubated with micrococcal nuclease at different concentrations of ox bile. The extent of digestion of human DNA by micrococcal nuclease was compared, as shown in Figure 2. The result showed that micrococcal nuclease remained active at the bile concentration up to 9%.

### Selective target DNA enrichment methods reduce the background human DNA prepared from blood samples

**Figure 3:**
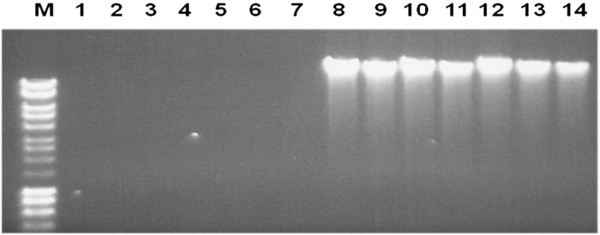
**DNA preparations made by STEM1 and the conventional method.** M: DNA marker; 1–7: DNA prepared by the STEM1; 8–14: DNA prepared by the conventional method. Lanes 1 and 8, 2 and 9, 3 and 10, 4 and 11, 5 and 12, 6 and 13, 7 and 14: DNA prepared from the spiked blood samples containing 3x10^5^, 3x10^4^, 3x10^3^, 3x10^2^, 30 , 3, 0 CFU/ml of *Salmonella* Typhi, respectively.

DNA prepared by the 2 different STEMs was compared with that isolated through the conventional method from blood samples spiked with *Salmonella* Typhi, as shown in Figure 3. There was a high background caused by human DNA in the preparations made through the conventional method (lanes 8–14), but not in the preparations made by STEM1 (lanes 1–7). Similar results were also obtained with STEM2 (data not shown).

### PCR sensitivity for detection of *Salmonella* Typhi is enhanced through the selective removal of background human DNA from blood samples

**Figure 4:**
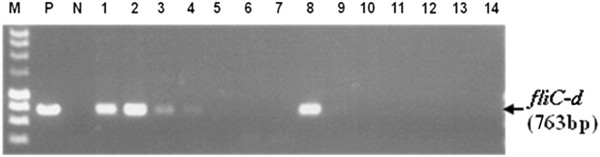
**Comparison of PCR sensitivities between the DNA preparations.***Salmonella* serovar Typhi *fliC-d* amplicons (763 bp) amplified by PCR using different DNA preparations were separated on a 1% agarose gel. M: DNA marker; P:* Salmonella* Typhi DNA positive control; N: No DNA negative control; Lanes 1–7: PCR on DNA prepared by STEM1; Lanes 8–14; PCR on DNA prepared by the conventional method. Lanes 1 and 8, 2 and 9, 3 and 10, 4 and 11, 5 and 12, 6 and 13, 7 and 14: DNA prepared from the spiked blood samples containing 3×10^5^, 3×10^4^, 3×10^3^, 3×10^2^, 30, 3, 0 CFU/ml of *Salmonella* Typhi, respectively.

The sensitivity of PCR was compared using DNA prepared by STEM and by conventional methods from the spiked blood samples. Figure 4 shows the *fliC-d* amplicons from the different DNA preparations. Using the DNA prepared with the conventional method, the *fliC-d* amplicons were only seen in the sample with 3x10^5^ CFU per millilitre of blood (equivalent to approximately 6,000 CFU per PCR). However, the *fliC-d* amplicons were seen in all the samples with 3x10^5^, 3x10^4^, 3x10^3^ and 3x10^2^ CFU per millilitre of blood, with highest sensitivity equal to approximately 6 CFU per PCR reaction. Independent experiments consistently found that STEM enhanced PCR sensitivity by 1,000 fold through selective removal of background human DNA in the spiked blood samples.

## Discussion

We describe a novel method (STEM) that enhances detection of *Salmonella* Typhi in spiked blood specimens. Using this method we were able to show a substantial reduction in dominating human background DNA in clinical samples and consequently increased the sensitivity of PCR detection of *Salmonella* Typhi from blood samples by 1,000 fold.

Early diagnosis and prompt treatment are essential for optimal management of typhoid patients, and therefore a sensitive and specific PCR assay is useful. Although many *Salmonella* Typhi PCR-based assays were studied in last decades, none has been brought to clinical use due to the limitations caused by low bactereamic level in typhoid patients. PCR sensitivity is directly related to the actual number of colony forming units found in the blood [3]. Typical clinical typhoid samples have <1 bacteria/ml of blood [11,19]. As a result, PCR offers only limited potential for typhoid diagnostics unless a new sample preparation method is developed. A DNA or bacterial capture system or even a culture enrichment step prior to amplification could improve PCR sensitivity. Using a culture enrichment step prior to amplification Teh *et al*. demonstrated that the 5 hour broth culture enrichment improved PCR sensitivity by 10 times for spiked blood, and 100 times for spiked stool samples [23]. We have recently developed a blood culture PCR assay which could detect as few as 0.75 bacteria per millilitre of blood following 3 hours of culture enrichment [22]. But like conventional blood culture, the PCR assay following bacterial culture enrichment still has some practical limitations including availability of culture facilities in disease endemic areas and bacteria being unculturable due to the use of antibiotics. Therefore, a PCR assay method that can be used directly on clinical blood samples is still a better choice. Collecting and then extracting DNA from a large volume of blood may theoretically improve PCR assays, but the presence of dominating human DNA causes false-positive PCR signals due to the non-specific binding of primers and false-negative results due to reduced sensitivity. Currently two protocols have been commercially marketed for reducing the presence of dominating human DNA:MolYsis (Molzym GmbH & Co. KG, Bremen, Germany) and Pureprove (SIRS-Lab GmbH, Jena, Germany). The former uses a chaotropic buffer containing guanidine hydrochloride to selectively lyze human cells. The released eukaryotic DNA was subsequently degraded by the addition of a chaotropic resistant nuclease, and the intact bacterial cells were collected for bacterial DNA purification. For the latter, total genomic (i.e. human and bacterial) DNA is first conventionally extracted specific bacterial genomic DNA is then isolated using a DNA binding protein that recognizes unmethylated CpG motifs predominantly present in bacterial and fungal genomes at significantly lower frequencies than in human genomes. Horz *et al.* recently compared these two protocols for DNA preparation, and found that both were able to substantially reduce the human background DNA in most of the cases but loss of bacterial DNA was also a problem [24]. For example, with MolYsis the recovery of *P. gingivalis* in periodontal samples was less than 20%. As mentioned above, the MolYsis protocol uses a guanidine hydrochloride chaotropic buffer for lysis of human/animal cells, and bacteria with a thin or labile cell wall (e.g. members of the genus *Treponema*) or those exposed to cell wall-active antibiotics and/or human immunosystems or bacteria even devoid of a cell wall (e.g. *Mycoplasma*, *Chlamydia*) are also lyzed. Therefore, the MolYsis protocol may not be suitable for use in the diagnosis of Gram-negative sepsis. They also found, on the other hand, the Pureprove technology was not efficient in removal of human DNA. This is because it is based on selective binding of bacterial DNA to a DNA binding protein that recognizes unmethylated CpG motifs. CpG islands and motifs are not distributed equally over the entire human genome, and fragments without any 5′-methylcytosin are present in every preparation and will mix with prokaryotic DNA. In addition, as cytosine methylation is negatively correlated with gene expression, in regions of active genes the CpG dinucleotides-motifs are also de-methylated and, if fragmentized, could possibly mix with prokaryotic DNA as well. It should be emphasized that in Horz *et al.* study, the mean absolute bacterial gene copy numbers was 2.23×10^10^ (saliva and supragingival plaque collected by cotton tampon) and 1.37x10^10^ (pooled subgingivalsulcus fluid) [24]. Such a high level of bacterial template in a blood sample from a patient with typhoid is unlikely. Therefore, the fact that typical clinical typhoid samples contain <1 organism/ml of blood highlights the big challenges faced for developing PCR diagnostics for typhoid.

Bile is a widely used ingredient in culture media for blood culture and the bile resistance of *Salmonella* is well-known. This could be exploited for developing typhoid diagnostics as bile can selectively lyze human blood cells and release both human DNA and intracellular bacteria without damaging bacterial cells [22,25]. In the present study, we have investigated the use of ox bile and micrococcal nuclease for selective lysis of blood cells and removal of background human DNA. We tested the use of micrococcal nuclease for removal of the released human DNA in the presence and absence of ox bile, and found that micrococcal nuclease is bile resistant and active at a wide range of bile concentrations used up to 9%. This suggests that micrococcal nuclease could be used to degrade human DNA while ox bile is added for the lysis of blood cells, potentially simplifying the design of typhoid diagnostics. We have tried two STEM protocols for removal of background human DNA and confirmed that both methods can substantially reduce background human DNA in the DNA preparations. As a result, the percentage of bacterial DNA in the preparation was increased. When the DNA prepared by STEM was used for PCR assay, we have consistently found that PCR sensitivity increased by at least 1,000 fold, compared with that using conventionally prepared DNA (which contained a large amount of background human DNA). These promising results indicate that STEM should now be investigated in field trials and to this end the approach is currently being applied in an endemic setting. Although the present study aims to improve current diagnostics for typhoid, the method described herein can be generally applied to sample preparation for diagnosis of infections with other ox bile resistant bacterial and fungal pathogens, in particular, where the low level of a pathogen presents a problem for current molecular diagnostics.

In conclusion, we describe herein a novel method, which we have termed “the selective target DNA enrichment method” (STEM). STEM can enrich bacteria from large volume samples, remove background human DNA, and enhance the sensitivity of PCR detection for *Salmonella* Typhi in blood samples. Therefore, this novel method of sample preparation offers a better option for improved typhoid PCR assays directly using clinical specimens in diagnosis of this globally important infection disease which we believe could be of importance in improving clinical care and providing effective evaluation of novel vaccines.

## Competing interests

LZ and AJP are authors on patents in the field of typhoid diagnostics.

## Authors' contributions

LZ and AJP conceived of the study and carried out its design. LZ performed the assays and drafted the manuscript. AJP edited the manuscript. All authors read and approved the final manuscript.

## Pre-publication history

The pre-publication history for this paper can be accessed here:

http://www.biomedcentral.com/1471-2334/12/164/prepub
